# Effects of nutritional supplement and resistance training for sarcopenia in patients with inflammatory bowel disease: A randomized controlled trial

**DOI:** 10.1097/MD.0000000000030386

**Published:** 2022-08-26

**Authors:** Jiaxi Zhao, Yiqin Huang, Xiaofeng Yu

**Affiliations:** a Department of Gastroenterology, Huadong Hospital Affiliated to Fudan University, Shanghai, People’s Republic of China.

**Keywords:** dietary supplements, inflammatory bowel disease, resistance training, sarcopenia, whey protein

## Abstract

Nutritional supplementation and resistance training are broadly recommended as part of the treatment of sarcopenia, but studies that have evaluated interventions in inflammatory bowel disease patients with sarcopenia are lacking. The aim of this study was to evaluate the effects of nutritional supplementation and resistance training for improving height-adjusted appendicular skeletal muscle mass (ASM/H^2^) and medical indices in patients with inflammatory bowel disease.

**Methods:**

This randomized, double-blind, placebo-controlled trial of forty-five participants was performed at Huadong Hospital Affiliated to Fudan University in Shanghai from September 2020 to June 2021. Eligible participants were randomly assigned to receive whey protein (10 g/d) or placebo (10 g/d) for 8 weeks while completing a resistance training program (3 times a week). Data such as ASM/H^2^ and other medical indices were collected at baseline and at 4 and 8 weeks of intervention.

**Results:**

Fifteen participants were allocated to the resistance training and whey protein (RT+WP) group, and thirteen participants were allocated to the resistance training and placebo (RT+placebo) group. The ASM/H^2^ significantly increased in the RT+WP group after 4 and 8 weeks of intervention, and the ASM/H^2^ of the RT+WP group was significantly higher than that of the RT+placebo group after 4 and 8 weeks of intervention (*F* = 1.092, *P* = .035). Both interventions significantly increased albumin (*F* = 7.214, *P* = .003). Hemoglobin and creatinine significantly increased in the RT+WP group (*F* = 3.592, *P* = .035; *F* = 3.922, *P* = .033, respectively). In addition, a significant group × time interaction was not observed for body mass index, 5-time chair stand test time, 3-metre walk speed, grip strength, waist circumference, hip circumference, or waist-to-hip ratio (*P* > .05).

**Conclusions:**

Nutritional supplementation may be effective in improving sarcopenia, as well as many other physiological indicators during resistance training.

## 1. Introduction

Sarcopenia, which is defined as an age-related decline in muscle mass and/or muscle function, places a huge burden on both individuals and society. It increases the risk of falls and fractures, causing mortality and morbidity.^[1]^ Therefore, reversing sarcopenia is significant for maintaining the health of the population. Sarcopenia is usually divided into 2 types: primary sarcopenia and secondary sarcopenia.^[2]^ Primary sarcopenia is often associated with age, while secondary sarcopenia is caused by one or more other obvious factors, such as insufficient activity, malnutrition, or systemic diseases.

Inflammatory bowel disease (IBD) is a chronic inflammatory disease of the gastrointestinal tract characterized by remission and relapse, mainly including Crohn’s disease (CD) and ulcerative colitis (UC). Studies have shown that compared with healthy controls, the skeletal muscle mass of IBD patients is significantly reduced, and the incidence of sarcopenia in IBD patients fluctuates between 36.7% and 65%.^[3]^ IBD patients are prone to sarcopenia, and sarcopenia might, in turn, increase their operation rate and postoperative complication occurrence rate, prolong their length of hospitalization and increase its cost,^[4]^ damaging the prognosis and quality of life of IBD patients.^[5–7]^

The main mechanisms for IBD causing sarcopenia are as follows.^[8]^ First, reduced food intake is due to various gastrointestinal symptoms, such as abdominal pain and diarrhea. Second, certain drugs, such as glucocorticoids, are often used. Third, an increase in body energy consumption is caused by chronic inflammation. Fourth, intestinal epithelial integrity and material transport function are impaired. Furthermore, disorders of the gut-muscle axis may also cause sarcopenia.^[9]^

Researchers have found that nutritional supplementation and resistance training are important interventions for sarcopenia.^[10–12]^ Resistance exercise can improve muscle strength and muscle function and promote muscle remodelling.^[13]^ Nutritional treatment includes protein supplementation, essential amino acids, vitamin D, bisphosphonates, calcifediol, and calcium. Among them, the effect of high-protein nutritional supplements (such as whey protein and collagen peptide) on sarcopenia has been confirmed to be effective.^[14,15]^ Nutrition and exercise together might be more beneficial to sarcopenia than nutrition or exercise alone. However, the basic nutritional status of patients with sarcopenia, the type, and dosage of protein supplements, and the type and frequency of exercise will have varying effects on sarcopenia. There are still a lack of studies on sarcopenia in patients with inflammatory bowel disease.

The most commonly used nutritional supplement in previous studies was whey protein.^[14,15]^ Whey protein is a protein derived from milk with high nutritional value and unique functional properties. Previous studies have shown that whey protein had the advantages of anti-inflammatory, immunomodulatory, protecting the intestinal mucosal barrier, improving muscle strength, and body composition.^[16,17]^ IBD patients may be intolerant to milk, but high-purity whey protein extracted from milk is often tolerated.

The guideline by Asian Working Group for Sarcopenia (AWGS) in 2019 used in this article is the latest diagnostic guide specified for the special physiological characteristics of the Asian population. This study is the first known intervention study using AWGS2019 for inflammatory bowel disease patients with sarcopenia.

## 2. Methods

### 2.1. Study design

This randomized, double-blind, placebo-controlled trial was performed in Huadong Hospital Affiliated with Fudan University from September 2020 to June 2021. The study was conducted in accordance with the Declaration of Helsinki and applicable local regulations and was approved by the Independent Ethics Committee of Huadong Hospital Affiliated to Fudan University (ethical number 20210129007). All participants signed an informed consent form before entering the trial.

### 2.2. Participants

**Figure 1.**
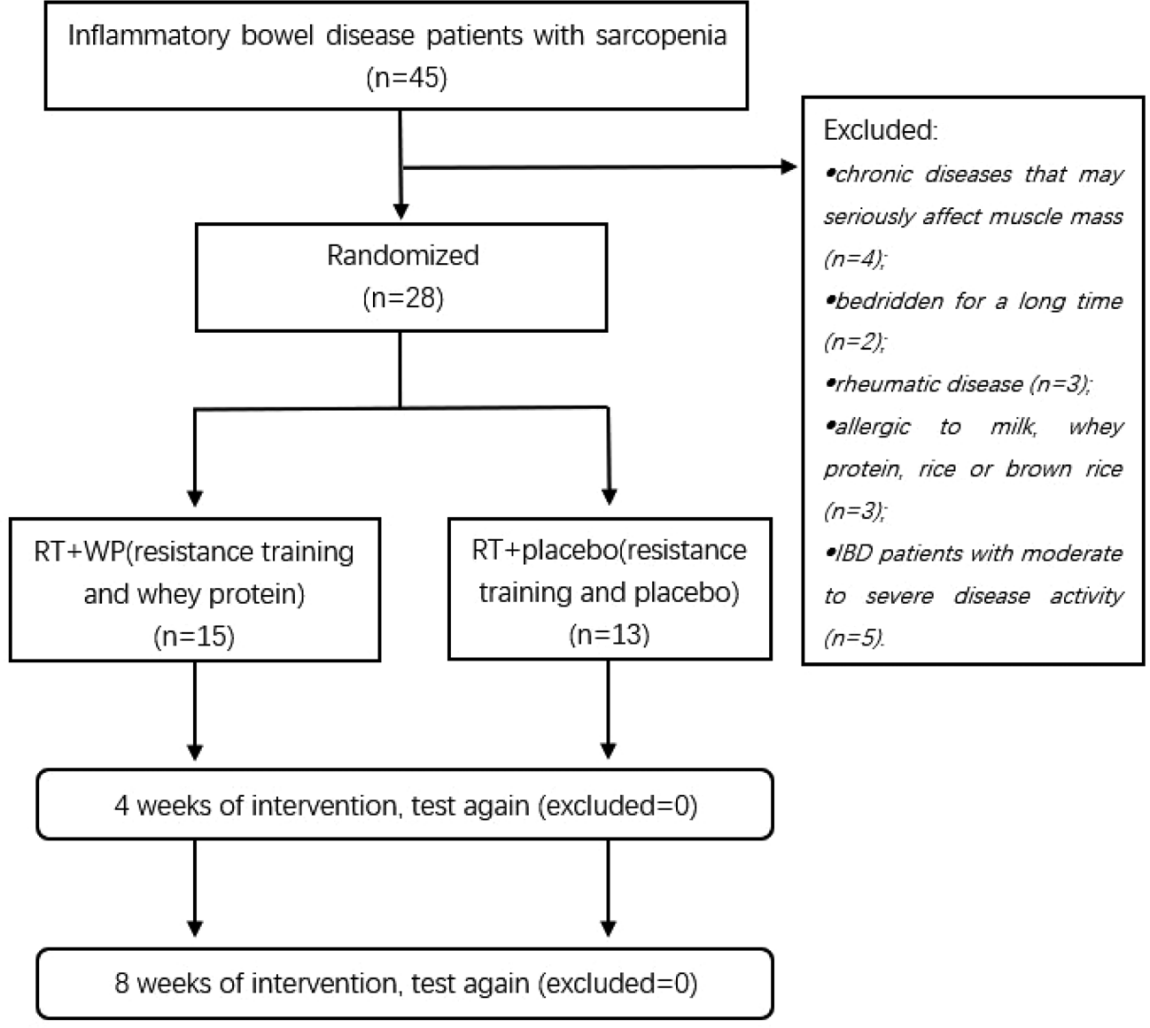
Flow chart of participant selection.

A total of 45 patients with IBD were diagnosed with sarcopenia by the AWGS2019 criteria.^[2]^ The inclusion criteria were as follows: aged between 18 and 65 years; daily total energy, protein, carbohydrate, and fat intake that had reached the Dietary Reference Intakes for Chinese (DRIs); nutritional status was in a low nutritional risk state after being screened by the Malnutrition Universal Screening Tool (MUST)^[18]^; and the intake of whey protein is <3 g/d. The exclusion criteria were as follows: patients with chronic diseases that may seriously affect muscle mass, such as chronic heart disease [n = 4]; patients who were bedridden for various reasons for a long time [n = 2]; patients with rheumatic disease [n = 3]; patients who were allergic to milk, whey protein, rice, or brown rice [n = 3]; and IBD patients with moderate to severe disease activity [n = 5]. After selection, 28 IBD patients with sarcopenia were included in the final intervention trial (Fig. 1).

### 2.3. Randomization and masking

The 28 participants that met the inclusion and exclusion criteria were randomly divided into 2 groups: RT+WP (resistance training and whey protein) group (n = 15) and RT+placebo (resistance training and placebo) group (n = 13). The RT+WP group consumed whey protein (10 g/d) and undertook resistance exercise training (3 times/wk), and the RT+placebo group consumed a placebo (10 g/d) and undertook resistance exercise training (3 times/wk). The placebo had the same appearance characteristics and calories as the whey protein group, and they all used the same packaging. We used rice paste powder in the placebo. To ensure that the treatment allocation was unbiased and concealed from the patients and researchers, randomization was performed by random codes generated by a computer. During the study period, all of the participants and researchers remained unaware of treatment allocation.

### 2.4. Procedures

Before the intervention, age, sex, height, weight, calf/waist/hip circumference, grip strength, disease type, duration of disease, medication, and severity of disease activity (Mayo scoring system Score for UC,^[19]^ Crohn’s Disease Activity Index (CDAI) for CD^[20]^) were collected by face-to-face systemic surveys before the intervention. Then, we calculated the 5-time chair stand test time, 3-metre walking speed, BMI, waist-to-hip ratio, and height-adjusted appendicular skeletal muscle mass (ASM/H^2^). The study used bioelectrical impedance analysis (BIA) to measure appendicular skeletal muscle mass. Hemoglobin, creatinine, ESR, C-reactive protein, and albumin were collected from venous blood after fasting for at least 8 hours. After 4 and 8 weeks of intervention, the above information was measured and collected again, and the ASM/H^2^ was measured again using BIA.

The Mayo scoring system for UC is based on only 5 items: A. stool frequency (normal stool frequency 1 = 1–2 times/d, 2 = 3–4 times/d, 3 = 5 times/d), B. rectal bleeding (0 = no rectal bleeding, 1 = mixed blood in the stool less than half the time, 2 = mixed blood in the stool most of the time, 3 = bleeding all the time), C. patient’s functional assessment score (0 = generally well, 1 = mild, 2 = moderate, 3 = severe), D. endoscopy findings (0 = normal, 1 = mild lesions (erythema, reduced blood vessel texture, mild fragility), 2 = moderate disease (obvious erythema, lack of blood vessel texture, fragility, erosion), and 3 = severe disease (spontaneous bleeding, ulcer formation)). A total score ≤2 points and no single subitem score >1 indicated clinical remission, 3 to 5 indicated mild activity, 6 to 10 indicated moderate activity, and 11 to 12 indicated severe activity.

The CDAI for CD is based on 5 items: A. general wellbeing (0 = very well, l = slightly below normal, 2 = poor, 3 = very poor, 4 = terrible). B. Abdominal pain (0 = none, 1 = mild, 2 = moderate, 3 = severe). C. number of liquid stools per day. D. Abdominal mass (0 = none, l = dubious, 2 = definite, 3 = definite and tender). E. complications: arthralgia, uveitis, erythema nodosum, aphthous ulcers, pyoderma gang. A total score ≤4 indicates a remission period, 5 to 7 indicates a light activity period, 8 to 16 indicates moderate activity and >16 indicates severe activity. Participants were instructed to dissolve 10 g of whey protein or placebo into 100 to150 mL water at a temperature lower than 40°C and consume it in the morning after breakfast.

The resistance exercise training instructional video was provided by the researchers to the participants. Before the resistance exercises, participants needed to perform 5 to 10 minutes of warm-up activities (outdoor brisk walking or indoor fast walking in place) and after the resistance exercises, they needed to perform 5 to 10 minutes of cooling down activities (thigh or calf stretching exercises). Resistance exercises were divided into half squats, back leg lifts, side leg lifts, lunges, and standing push-ups. Participants were asked to repeat each action 20 to 30 times. The whole resistance exercise training was repeated twice.

Subjects who did not complete the follow-up or have intolerance or worsening symptoms of inflammatory bowel disease (abdominal pain, diarrhea, blood in the stool, etc^[21]^) during participation in the trial will not be included in the final statistical analysis.

### 2.5. Outcomes

The primary outcome was ASM/H^2^; the secondary outcomes were blood tests (hemoglobin, creatinine, erythrocyte sedimentation rate (ESR), C-reactive protein and albumin), BMI, 5-time chair stand test, 3-metre walking speed, grip strength, calf/waist/hip circumference, and waist-to-hip ratio.

### 2.6. Assessment of skeletal muscle mass

In this study, an Inbody body composition analyzer was used to measure appendicular skeletal muscle mass. During the measurement, the patients were instructed to fast, defecate, urinate, wear light clothes, and undergo the measurement before exercising, bathing, and showering. Female patients should avoid being measuring during menstrual periods. We took the measurements in the same time period and in the same environment to keep the temperature (10–40°C) and humidity (10–95% Relative Humidity) of the environment relatively stable. Then, the ASM/H^2^ was calculated.

### 2.7. Data analysis

SPSS 26 was used for statistical analysis. Normally distributed continuous variables were represented by $\bar{x}\pm \text{s}$, and nonnormally distributed continuous variables were represented by M (P25, P75). Two-way repeated-measures ANOVA (intervention × duration) was used to evaluate the interventions of whey protein and resistance exercise. Then, we performed a simple main effect on variables that showed significant differences to determine the differences between the groups. The differences between groups were analyzed by *t* tests. Categorical variables were expressed as percentages, and differences between groups were analyzed using the χ^2^ test or Fisher’s exact probability method. *P* < .05 was considered statistically significant.

## 3. Results

All 28 participants completed the entire follow-up process of the experiment. These 28 sarcopenia patients were evaluated using AWGS2019. The average age of the IBD patients with sarcopenia was 44.1 years, and the male-to-female ratio was 19/9. The average BMI was 21.2 kg/m^2^, and the average grip strength was 34.87 kg. For calf/waist/hip circumference, the average values were 34.5, 79.3, and 93 cm, respectively, and the mean waist-to-hip ratio was 0.85. For the exercise tests of physical performance, the average 5-time chair stand test time was 6.8 seconds, and the median 3-metre walk speed was 1.05 m/s.

Of the 28 patients, 15 were allocated to the RT+WP group and 13 to the RT+placebo group. There were no differences between the RT+WP group and RT+placebo group at baseline (Table 1).

**Table 1:** The characteristics of clinical and anthropometric measurements of the participants.

Variables	Group RT+WP	Group RT+placebo	*P* value
Study population	15	13	NA
Age (yr)	45.7 ± 13.0	42.2 ± 12.1	.444^*^
Male/female	11/4	8/5	.689^†^
BMI (kg/m^2^)	21.6 ± 3.1	20.7 ± 2.2	.367^*^
Grip strength (kg)	36.7 ± 10.8	31.7 ± 12.6	.304^*^
Calf circumference (cm)	35.3 ± 3.6	33.5 ± 3.5	.177^*^
Waist circumference (cm)	81.1 ± 8.8	74.1 ± 12.6	.096^*^
Hip circumference (cm)	93.8 ± 4.1	91.3 ± 3.9	.109^*^
Waist to hip ratio	0.87 ± 0.09	0.81 ± 0.12	.169^*^
Smoking, n (%)	4 (26.7%)	2 (15.4%)	.655^*^
Drinking, n (%)	0	0	NA
5-time chair stand test (s)	7.0 ± 1.5	6.6 ± 1.6	.573^*^
3-m walk (m/s)	1.0 ± 0.3	1.1 ± 0.2	.457^*^
Duration of disease (mo)	54.4 ± 74.4	57.9 ± 67.6	.898^*^
Severity of activity (m1/m2/m3/m4)	2/8/4/1	1/4/5/3	.489^†^
Glucocorticoid use (n1/n2/n3)	10/4/1	9/0/4	.082^†^
Hemoglobin (g/L)	131.8 ± 18.3	132.8 ± 19.8	.894^*^
Creatinine (µmol/L)	64.0 ± 10.9	65.2 ± 11.6	.792^*^
Erythrocyte sedimentation rate (mm/h)	20.9 ± 19.8	18.6 ± 7.9	.47^*^
C-reactive protein (mg/L)	7.3 ± 11.8	4.7 ± 5.1	.481^*^
Albumin (g/L)	38.0 ± 5.1	39.4 ± 5.0	.457^*^

BMI = body mass index, m1 = remission, m2 = mild, m3 = moderate, m4 = severe (Mayo scoring system or CDAI), n1 = <28 d, n2 = 28 d to 3 mo, n3 = >3 mo, NA = not available, RT+WP = resistance training and whey protein.

* *t* test.

† Fisher’s exact probability method.

### 3.1. ASM/H^2^

**Figure 2.**
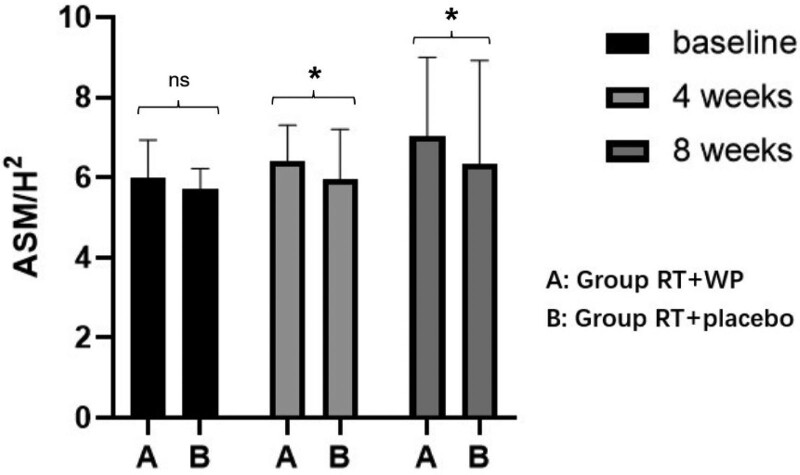
Comparison of ASM/H^2^ results of the 2 groups. ASM/H^2^ = height-adjusted appendicular skeletal muscle mass.

There was a significant group × time interaction for ASM/H^2^ (*F* = 1.092, *P* = .035; 4-weeks > baseline [Group RT+WP]; 8 weeks >baseline [Group RT+WP]; Group RT+WP > Group RT+placebo [4 weeks]; Group RT+WP > Group RT+placebo [8 weeks]) (Fig. 2; Table 2).

**Table 2:** Comparison of ASM/H^2^ of the 2 groups.

Duration	Variables	Group RT+WP	Group RT+placebo	*F* value for intervention × duration, *P* value	Simple main effect
Baseline	ASM/H^2^ (kg/m^2^)	5.98 ± 0.52	5.71 ± 0.34	1.092, .035	4 wk > baseline
4 wk		6.41 ± 0.58	5.96 ± 0.47		(Group RT+WP)
8 wk		7.03 ± 0.74	6.33 ± 0.91		8 wk > baseline
					(Group RT+WP)
					Group RT+WP > Group RT+placebo (4 wk)
					Group RT+WP > Group RT+placebo (8 wk)

ASM/H^2^ = height-adjusted appendicular skeletal muscle mass, NA = not available, RT+WP = resistance training and whey protein.

### 3.2. Blood tests

There was a significant group × time interaction for hemoglobin (*F* = 3.592, *P* = .035; 4 weeks > baseline [Group RT+WP]; 8 weeks > baseline [Group RT+WP]; Group RT+WP > Group RT+placebo [8 weeks]), creatinine (*F* = 3.922, *P* = .033; 4 weeks > baseline [Group RT+WP]; 8 weeks > baseline [Group RT+WP]; Group RT+WP > Group RT+placebo [4 weeks]; Group RT +WP>Group RT+placebo [8 weeks]) and albumin (*F* = 7.214, *P* = .003; 4 weeks > baseline [Group RT+WP, Group RT+placebo]; 8 weeks > baseline [Group RT+WP, Group RT+placebo]; 8 weeks > 4 weeks [Group RT+WP, Group RT+placebo]; Group RT+WP > Group RT+placebo [8 weeks]). There was no significant group × time interaction for ESR and C-reactive protein (Table 3).

**Table 3:** Comparison of laboratory test results of the 2 groups.

Duration	Variables	Group RT+WP	Group RT+placebo	*F* value for intervention × duration, *P* value	Simple main effect
Baseline	Hemoglobin (g/L)	131.8 ± 18.3	132.8 ± 19.8	3.592, .035	4 wk > baseline
4 wk		141.0 ± 10.8	132.4 ± 17.6		(Group RT+WP)
8 wk		143.9 ± 9.7	132.0 ± 14.6		8 wk > baseline
					(Group RT+WP)
					Group RT+WP > Group RT+placebo (8 wk)
Baseline	Creatinine (μmoI/L)	64.0 ± 10.9	65.2 ± 11.6	3.922, .033	4 wk > baseline
4 wk		72.1 ± 7.4	65.5 ± 8.4		(Group RT+WP)
8 wk		74.7 ± 7.2	66.8 ± 8.5		8 wk > baseline
					(Group RT+WP)
					Group RT+WP > Group RT+placebo (4 wk)
					Group RT+WP>Group RT+placebo (8 wk)
Baseline	Erythrocyte sedimentation rate (mm/h)	20.9 ± 19.8	18.6 ± 7.9	0.800, .461	NA
4 wk		16.7 ± 13.8	8.2 ± 7.2		
8 wk		14.5 ± 13.6	7.2 ± 7.0		
Baseline	C-reactive protein (mg/L)	7.3 ± 11.8	4.7 ± 5.1	0.635, .538	NA
4 wk		2.2 ± 1.1	3.1 ± 3.2		
8 wk		2.9 ± 4.6	2.7 ± 2.9		
Baseline	Albumin (g/L)	38.0 ± 5.1	39.4 ± 5.0	7.214, .003	4 wk > baseline
4 wk		43.2 ± 3.8	40.2 ± 4.9		(Group RT+WP, Group RT+placebo)
8 wk		45.2 ± 3.1	42.0 ± 5.0		8 wk > baseline
					(Group RT+WP, Group RT+placebo)
					8 wk > 4 wk
					(Group RT+WP, Group RT+placebo)
					Group RT+WP > Group RT+placebo (8 wk)

NA = not available, RT+WP = resistance training and whey protein.

### 3.3. BMI, 5-time chair stand test, 3-metre walk and grip strength

There was no significant group × time interaction for BMI, 5-time chair stand test, 3-metre walk or grip strength (Table 4).

**Table 4:** Comparison of body mass index, 5-time chair stand test, 3-metre walk and grip strength of the 2 groups.

Duration	Variables	Group RT+WP	Group RT+placebo	*F* value for intervention × duration, *P* value	Simple main effect
Baseline	BMI (kg/m^2^)	21.6 ± 3.1	20.7 ± 2.2	3.059, .065	NA
4 wk		22.6 ± 3.0	20.4 ± 2.3		
8 wk		23.2 ± 3.1	20.9 ± 2.1		
Baseline	5-time chair stand test (s)	7.0 ± 1.5	6.6 ± 1.6	1.231, .309	NA
4 wk		6.7 ± 1.4	6.4 ± 1.5		
8 wk		6.2 ± 1.4	6.2 ± 1.3		
Baseline	3-m walk (m/s)	1.0 ± 0.3	1.1 ± 0.2	1.001, .382	NA
4 wk		0.9 ± 0.2	1.0 ± 0.2		
8 wk		0.9 ± 0.1	1.0 ± 0.2		
Baseline	Grip strength (kg)	36.7 ± 10.8	31.7 ± 12.6	3.184, .059	NA
4 wk		41.5 ± 8.7	33.4 ± 14.0		
8 wk		42.6 ± 8.4	32.9 ± 12.5		

BMI = body mass index, NA = not available, RT+WP = resistance training and whey protein.

### 3.4. Calf/waist/hip circumference and waist to hip ratio

There was a significant group × time interaction for calf circumference (*F* = 8.220, *P* = .002; 4 weeks > baseline [Group RT+WP, Group RT+placebo]; 8 weeks > baseline [Group RT+placebo]). There was no significant group × time interaction for waist circumference, hip circumference or waist-to-hip ratio (Table 5).

**Table 5:** Comparison of calf/waist/hip circumference and waist to hip ratio of the 2 groups.

Duration	Variables	Group RT+WP	Group RT+placebo	*F* value for intervention × duration, *P* value	Simple main effect
Baseline	Calf circumference (cm)	35.3 ± 3.6	33.5 ± 3.5	8.220, .002	4 wk > baseline
4 wk		35.8 ± 3.7	34.7 ± 3.8		(Group RT+WP,Group RT+placebo)
8 wk		35.8 ± 3.7	36.3 ± 3.7		8 wk > baseline
					(Group RT+placebo)
Baseline	Waist circumference (cm)	81.1 ± 8.8	74.1 ± 12.6	0.295, .747	NA
4 wk		82.8 ± 8.7	78.8 ± 8.0		
8 wk		84.0 ± 8.5	79.6 ± 7.9		
Baseline	Hip circumference (cm)	93.8 ± 4.1	91.3 ± 3.9	1.346, .276	NA
4 wk		94.7 ± 4.0	91.8 ± 3.9		
8 wk		95.1 ± 4.0	93.3 ± 5.8		
Baseline	Waist to hip ratio	0.87 ± 0.09	0.81 ± 0.12	1.691, .205	NA
4 wk		0.88 ± 0.09	0.85 ± 0.67		
8 wk		0.88 ± 0.08	0.85 ± 0.06		

NA = not available, RT+WP = resistance training and whey protein.

## 4. Discussion

This study is the first known intervention study using AWGS2019 for IBD patients with sarcopenia. Nutritional supplementation may be effective in improving sarcopenia as well as many other physiological indicators during resistance training. The indicators in this study included ASM/H^2^, hemoglobin, creatinine, albumin, and calf circumference.

The results of this study showed that both interventions improved ASM/H^2^, but after 4 or 8 weeks of intervention, the ASM/H^2^ of the nutritional supplementation combined resistance training group was higher than that of the resistance training group. These results are in line with the previous findings. The effects of exercise and nutrition on sarcopenia have been proven in previous studies. Hun Kyung Kim’s research showed that exercise combined with amino acid supplementation could effectively increase muscle mass, muscle strength, and physical function in sarcopenia patients.^[22]^ However, Bonnefoy et al^[23]^ pointed out that exercise and nutrition could improve muscle function but had no significant effect on muscle mass, and Tieland et al^[24]^ MSc pointed out that protein supplementation could improve body functions, but it might not increase muscle mass in elderly sarcopenia patients. Exercise can improve muscle mass by increasing the expression of insulin-like growth factor 1 (IGF-1),^[22]^ and resistance training is the most effective exercise method to increase muscle mass. Protein is the raw material for muscle synthesis, and proteins that are rich in essential amino acids may improve the muscle mass and strength of sarcopenia patients.^[25]^ A comprehensive treatment with nutrition and exercise is more beneficial to sarcopenia than nutrition or exercise alone.

In the present study, creatinine after 4 weeks of nutritional supplementation combined with resistance training was higher than that after resistance training alone, and hemoglobin, creatinine, and albumin after 8 weeks of nutritional supplementation combined with resistance training were higher than those after resistance training alone. Furthermore, both interventions increased albumin. Nutritional supplementation combined with resistance training could increase hemoglobin and creatinine, but resistance training alone did not have an effect on albumin and creatinine. Neither intervention had an effect on ESR or C-reactive protein. These research results are consistent with previous studies. It is well known that albumin synthesis is stimulated by dietary amino acids, proteins, and exercise, especially proteins.^[26]^ Dietary amino acids and protein can increase muscle protein synthesis, thereby achieving a positive nitrogen balance.

Theoretically, nutritional supplementation combined with resistance training will reduce ESR and C-reactive protein, which has been confirmed in previous studies. A combination of the 2 interventions is better than an individual intervention.^[27]^ A systematic review showed that 8 weeks of high-intensity exercise could reduce C-reactive protein.^[28]^ However, an exercise intervention study for patients with chronic kidney disease showed that albumin and C-reactive protein did not change before and after exercise intervention.^[28]^ Muscle contraction caused by resistance training leads to the production of muscle cytokines. It has anti-inflammatory effects and can antagonize proinflammatory cytokines, reducing inflammation and lowering ESR and C-reactive protein.^[28]^ The heterogeneity between trials may be related to differences in the exercise intervention methods and the participants.

Previously, some researchers thought that BMI could be used to assess sarcopenia, but it has poor accuracy in reflecting the body composition of patients.^[29]^ Studies have shown that although the median BMI of patients with sarcopenia tends to be lower than that of healthy people, 40% of patients with sarcopenia had a normal BMI, and 20% of patients with sarcopenia were overweight or obese.^[30]^ The 5-time chair stand test, 3-metre walking speed, and grip strength are sensitive indicators reflecting skeletal muscle function, which are recommended by the AWGS2019 diagnostic criteria. In the present study, statistically significant changes in BMI, 5-time chair stand test time, 3-metre walking speed, and grip strength were not observed in either group. The different conclusions between the results of this study and previous studies might be due to differences in the intervention methods. For example, the frequency, intensity, and duration of resistance exercise training.

Bunout et al^[31]^ research results showed that resistance exercise could significantly improve the strength of the subjects’ quadriceps muscles and reduce the chair stand test time. At the same time, vitamin D and calcium supplementation can strengthen the above effects and increase walking speed. Bunot et al^[32]^ research pointed out that 2 times/wk of resistance exercise training could increase the lower limb strength, walking speed, and grip strength of patients. Brose et al^[33]^ research pointed out that resistance exercise training could safely increase the muscle strength and function of elderly individuals. Resistance exercise training improves muscle function by increasing mitochondrial respiration, ATP production, enzyme activity, and the protein content of muscle cells.^[34,35]^

In the present study, statistically significant changes in waist circumference, hip circumference, and waist-to-hip ratio were not observed in either group. However, the calf circumference of both groups after 4 weeks of intervention was greater than at baseline, and the calf circumference of 8 weeks of nutritional supplementation combined with resistance training intervention was greater than at baseline. In clinical practice, calf circumference measurement is a convenient way to roughly assess the skeletal muscle mass.^[36]^ It has high sensitivity in predicting sarcopenia and is especially suitable for primary health care institutions. AWGS2019 recommends the use of nonelastic tape to measure the maximum value of the 2 calf circumferences.^[2]^ In addition, the measurement of waist and hip circumference helps to screen for central obesity, which is usually defined as BMI ≥28 kg/m^2^ and a waist circumference >90 cm for men and >85 cm for women. This helps to screen for sarcopenic obesity, which is the coexistence of sarcopenia and obesity. As endocrine organs, adipocytes can secrete adipokines as active factors.^[37]^ The hypertrophy, proliferation, and activation of adipocytes trigger a disorder of adipokines and cytokines, thereby causing chronic inflammation in the body and promoting the occurrence of sarcopenia.^[38]^

This study also had a few limitations. First, the subjects were from one hospital, and consequently, the results may not be generalizable. However, this a typical general hospital in Shanghai and the participants are very representative of the general IBD population. Second, since the subjects of this study were all Asian, the diagnostic criterion of this study was AWGS2019, which may lack universality, but AWGS2019 has been widely used in Asian populations. Third, due to ethical reasons, this study cannot determine the effect of nutritional supplementation combined with resistance training intervention versus resistance training intervention alone on inflammatory bowel disease patients with sarcopenia.

In conclusion, the present study is the first to investigate the effects of nutritional supplementation and resistance training for sarcopenia in patients with inflammatory bowel disease in a randomized controlled trial. Nutritional supplementation combined with resistance training enhances ASM/H^2^, albumin, hemoglobin, creatinine, and calf circumference. Nutritional supplementation combined with resistance training is better than resistance training alone to enhance ASM/H^2^ over 4 and 8 weeks of intervention. Future studies should aim to replicate the results in a larger population. It is significant to investigate different interventions for sarcopenia in IBD patients in the future to improve the prognosis of IBD patients.

## Author contributions

**Conceptualization:** Jiaxi Zhao.

**Data curation:** Jiaxi Zhao.

**Resources:** Xiaofeng Yu.

**Investigation:** Yiqin Huang.

**Methodology:** Yiqin Huang.

**Supervision:** Xiaofeng Yu.

**Visualization:** Xiaofeng Yu.

**Writing – original draft:** Jiaxi Zhao.

**Writing – review & editing:** Jiaxi Zhao.
